# Public Health Decision-Maker Perspective on Joint Clinical Assessments in Central European EU Member States

**DOI:** 10.3390/jmahp13010010

**Published:** 2025-03-04

**Authors:** Gergő Merész, Dávid Dankó, Márk Péter Molnár

**Affiliations:** 1 MediConcept Ltd., H-1036 Budapest, Hungary; 2 Ideas & Solutions Ltd., H-1114 Budapest, Hungary; david.danko@i-s.hu (D.D.); mark.molnar@i-s.hu (M.P.M.)

**Keywords:** value-based healthcare, technology assessment, health, health policy, decision-making, organizational, joint clinical assessment

## Abstract

The HTA R introduces provisions that may benefit member states, in particular the opportunity to share national or regional assessment reports, cooperate outside of clinical domains, or use the methodological guidelines on a local level for technologies that are not subject to joint assessment. Challenges related to the timelines, differences between assessment scopes, and diverging guidance may jeopardize the full potential of the HTA R in Central European EU member states. However, these are more likely to be related to the commitment and vigilance of local competent authorities. We attempt to address these opportunities and mark some challenges imposed by the application of the HTA R by taking the perspective of public health decision-makers in Central European EU member states. We conclude that the foundations for capitalizing on the opportunities offered by the HTA R are already laid in the region, and we foresee policymakers and payers sharing the responsibility of acting as drivers of change in health policy to reduce the duplication (or multiplication) of efforts by HTDs, as well as to increase the efficient use of HTA bodies’ resources.

## 1. Introduction

Building a strong European Health Union has been on the policy agenda of the European Commission for several years, with the COVID-19 pandemic eventually giving a boost to delivering on priorities such as the accessibility and affordability of medicine [1]. The commitment of the member states, the European Parliament, and the European Commission is reflected by the fact that, following three years of negotiations and three preceding joint actions, Regulation (EU) 2021/2282 of the European Parliament and of the Council of 15 December 2021 on health technology assessment and amending Directive 2011/24/EU (in short, HTA R) was adopted. In brief terms, the HTA R foresees a process that starts with the Member State Coordination Group on Health Technology Assessment (HTACG), setting up a scope for the joint clinical assessment (JCA) of a technology, and the health technology developer (HTD) submitting evidence that fits within this scope. The HTACG then proceeds to produce and adopt a JCA Report according to a set of methodological guidelines. This report can be used by member states for their respective decision-making procedures [2]. The initial set of medicinal products subject to the JCA procedure consists of new active substances for the treatment of cancer as well as advanced therapy medicinal products. According to the HTA R, orphan medicinal products will be added to this set in January 2028; all centrally authorized medicinal products will be subject to JCAs from January 2030. The HTA R harbors additional elements that may benefit member states, such as the opportunity to share national or regional assessment reports, cooperate outside of clinical domains, or use the methodological guidelines on a local level for technologies that are not subject to joint assessment.

Considering the time and resources allocated to the preparatory efforts, including drafting and adopting the HTA R, as well as the potential benefits of increased cooperation and harmonization, the uptake of joint assessment products would be highly desirable. Since healthcare funding levels tend to be lower in Central Europe than in other member states of the European Union, decision-support frameworks should ideally be based on solid evidence, improve efficiency, and be efficient themselves. In this setting, the primary stakeholders are public health decision-makers of member states, who need to see tangible benefits from HTA R while containing risks arising from the adjustment of the local administrative processes at a reasonable level to enable the seamless use of JCAs. In this opinion paper, we set out to discuss these opportunities and challenges imposed by applying the HTA R by taking the perspective of public health decision-makers in Central European EU member states. To support the uptake of JCAs for pricing and reimbursement decisions, we hereby present a narrative review of sections of the HTA R that we see critical in enabling access to relevant and timely evidence, as well as for having the appropriate methodological guidance.

## 2. Opportunities

First, acquiring the relevant evidence for the decision problem might be difficult in some settings, even if the necessary pieces of evidence exist, but there are (for example, confidentiality) barriers to accessing it, whereas the same pieces of evidence are shared by HTDs with competent authorities operating on major markets. The HTA R addresses this in Article 13(1) point *e)*, stating that member states have to share all data received from the HTD locally that fit into the scope of the JCA. In practical terms, this would enable the access of any national or regional health technology assessment (HTA) body to a variety of analyses, including clinical study reports, on-file post hoc analyses and indirect comparisons, without having to rely either on specific inputs from the HTD’s local affiliate or its willingness to cooperate. This provision in the HTA R carries the potential to reduce the duplication (or multiplication) of efforts by HTDs, as well as to increase the efficient use of HTA bodies’ resources.

This opportunity has a potential synergistic effect with the ‘inclusiveness’ requirement in the scoping of assessments as per Article 8(6) of the HTA R, and the option to include a variety of analyses, especially information on studies based on registries in the submission as stated in Annex I(d). These two points in the HTA R together enable HTA bodies’ access to further analyses that can benefit decision-making. A key to harnessing these advantages is having a value framework in use at national or regional levels that makes it possible to conclude on the clinical added benefit [3]. Such value frameworks can be implemented to the assessment and appraisal procedure and have the potential to inform economic evaluation or translate the sources of uncertainty into arguments on fair pricing [4] and risk-sharing. An analysis of 269 reimbursement submissions in Hungary [5] found that the reimbursement decision shows a clear association with the HTA body’s conclusion of the existence of clinical added benefit, even after adjustment for multiple factors and despite the fact that demonstrating clinical added benefit is not an explicit criterion for reimbursement in Hungary.

The next opportunity for decision-makers is to have access to the assessment reports of competent authorities operating in other member states (see Article 23(5)). Such documents may serve as a basis for external validation exercises of the appraisal process and could contribute to the quality assurance of the HTA body’s deliverables. Moreover, the availability of national or regional assessment reports could foster collaborations in the Central European Region beyond the domain of clinical assessments. Similar cooperations of competent authorities already exist between Denmark, Finland, Iceland, Norway, and Sweden (Joint Nordic HTA Bodies) or between Belgium, the Netherlands, Luxemburg, Austria, and Ireland (the Beneluxa Initiative). Others have also discussed the role of such collaborations in the context of the HTA R [6]. Nevertheless, the primary motivation of HTA bodies for reviewing others’ assessments and appraisal reports remains their search for external reassurance on conclusions by entities that are void of conflicting interests. The precursors of such efforts to collaborate can already be traced in the methods manuals and submission templates of the HTA bodies. For example, the methods manual issued by the Ministry of Health and the National Institute for Value and Technologies in Healthcare in Slovakia specifically refers to including the conclusions of the assessments produced by other HTA bodies in Europe, highlighting the Czech Republic, Germany, and England, as well as France, Scotland, and, from outside Europe, Canada.

The final advantage we highlight here is the importance of state-of-the-art guidance on methods. Due to the lack of sufficient resources, HTA bodies in Central European EU member states may face challenges in internal knowledge management, as well as in providing guidance for HTDs and producers of reimbursement submissions. This phenomenon undermines the institutional embeddedness of evidence-based decision-making, also contributing to a focus shift towards fiscal aspects which are indirectly related to the core assessment procedure. If high-quality guidance, created through joint efforts by experts of HTA bodies for whom strict rules on conflicts of interest apply, becomes available, significant savings can be generated on method development exercises. This opportunity also has a potential synergistic effect by ensuring the seamless uptake of JCAs in local assessment procedures.

## 3. Challenges

Inevitably, there are some challenges, too, which may jeopardize the full potential of the HTA R in Central European EU member states, although these are more likely to be related to the commitment and vigilance of local competent authorities. Most importantly, the tight timelines related to setting up the assessment scope, which aims to help the timeliness of the JCA, is difficult to match, especially with the frequency of such exercises expected to grow as the range of technologies subject to the HTA R is set to broaden (see Article 7(2)). This will likely contribute to an increased interest of patients and clinical and other experts in being involved in setting up the local scope. The risk of not being able to meet the deadlines, or overlooking them, can be mitigated by the upfront development of local procedures and easy-to-use ‘stakeholder-friendly’ templates for setting the local scope. Others have already identified similar challenges affecting several stakeholders and proposed possible action points via qualitative exercises [7].

Another threat to realizing the benefits of the HTA R emerges from differences between the scope of the JCA and the local dossier compiled by the HTD. Such differences may ultimately lead to the limited uptake of the JCA, while efforts to generate the right scope and the contributions to producing the joint report have already all been made. To overcome this, HTA bodies should use the inclusive feature of the scoping process and consider including scenarios which are conditional upon the outcome of decisions on currently ongoing assessments. These decisions not only define the potential comparator technologies but can also delineate patient populations included in future assessments.

The last challenge we highlight here is the possibility of divergences and contradictions between local guidance and the guidance produced under the HTA R. Although the full alignment of guidance on clinical aspects would seem self-evident, there are very clear settings where there is a strong local interdependence between clinical, economic, and implementation domains. For example, the local guidance on economic evaluations suggests using authorized technologies as comparators as the base case, whereas the HTA R and the accompanying guidance emphasize the inclusive feature of the assessment scope. Choosing the relevant comparator can be influenced by reimbursement status and method, or the acceptability of evidence generated outside clinical study programs. To address this challenge to the largest possible extent, it is necessary to revise not only local guidance on clinical domains but also on economic domains.

Table 1 provides a summary of the key opportunities and challenges we identified from the perspective of public health decisionmakers in Central European EU member states.

## 4. Conclusions

To conclude from the perspective of former public health decision-makers in Central Europe, we wish to emphasize that the foundations for capitalizing on the opportunities offered by the HTA R are already laid in the region. From now on, policymakers and payers will be sharing the responsibility of acting as drivers of change in health policy and exploiting new opportunities for changing healthcare systems for the better.

## Figures and Tables

**Table 1:** Summary of key opportunities and challenges.

Key Opportunities	Key Challenges
Reducing duplication efforts for HTDs and increasing the efficient use HTA bodies’ resources	Adhering to procedural timelines as the number of technologies subject to joint clinical assessments keeps growing, particularly for setting up the assessment scope.
Enabling access of HTA bodies to a variety of analyses	Inconsistency between the scope of the JCA and the local reimbursement submission by the HTD.
Using assessment reports of other national competent authorities as a basis for external validation of findings and quality assurance	Divergence and contradiction between local guidance and the guidance produced under the HTA R.
Having state-of-the-art methodological guidance	
